# Combined use of remifentanil and propofol to limit patient movement during retinal detachment surgery under local anesthesia

**DOI:** 10.4103/1658-354X.71570

**Published:** 2010

**Authors:** Abdul Kader M. Mahfouz, Ashraf M. Ghali

**Affiliations:** *Department of Anaesthesia, Alexandria University Egypt*; 1 *Department of Anaesthesia, Tanta University, Egypt*

**Keywords:** *Eye surgery*, *propofol*, *regional anesthesia*, *remifentanil*, *sedation*

## Abstract

**Background:**

One of the drawbacks of performing ophthalmic surgery under local anesthesia is patient movement, which might affect optimal surgical outcome.

**Purpose:**

The study aims to evaluate the efficacy of the combined use of propofol and remifentanil as a sedative technique in comparison with the use of propofol alone to limit patient discomfort and movement during local anesthesia for vitreo-retinal surgery lasting for more than two hours.

**Materials and Methods:**

A total of 140 patients scheduled for vitreo-retinal surgery under local anesthesia, with an expected surgical time of more than two hours, were included in the study. Patients were divided randomly into two equal groups: group I where patients were given propofol and remifentanil by continuous infusion and group II where patients were given propofol alone by continuous infusion.

**Results:**

The two groups were comparable with regard to age, weight, gender, ASA physical status and duration of surgery. There was a significant decrease in heart rate and mean arterial blood pressure (MABP) in each group 10 minutes after the start of sedation compared with pre-sedation data and continued all through the procedure. There was an insignificant difference between the two groups with regard to changes in heart rate and MABP all through surgical procedure. There was no significant difference between the two groups with regard to the incidence of complications except for an increased incidence of breakthrough pain and discomfort which necessitated the use of fentanyl as a rescue treatment in the propofol group *P*<0.001. There were no instances of movements with a major effect on the surgical field, which could have affected surgical outcome, in the two groups. The number of patients who did not move was significantly higher, 56 (80%), in group I compared with 38 (54.29%) in group II with *P*<0.001. The ophthalmologist satisfaction scale was significantly higher in group I (4.5±0.63) compared with group II (3.7±1.04) with *P*=0.0016.

**Conclusion:**

The combined use of propofol and remifentanil as a continuous infusion before performance of the block and during lengthy vitreo-retinal surgery was associated with a lower incidence of patient discomfort, breakthrough pain, and patient movement along with high degree of surgeons’ satisfaction and hemodynamic stability.

## INTRODUCTION

Large numbers of patients undergoing ophthalmic surgery are elderly with multisystem diseases.[1] Many ophthalmic procedures can be performed safely in an outpatient setting, using local (peri-retrobulbar block) or topical anesthesia. Considerable drawbacks of local anesthesia in these patients include the fact that a few geriatric patients can remain comfortable on an operating table for procedures that exceed two or three hours.[2] According to the American Society of Anesthesiologists Closed Claim database, patient movement during ophthalmologic surgery was the second most common cause of eye injury associated with anesthesia.[3–4]

Conscious sedation during ocular surgery, especially lengthy procedures like vitreo-retinal surgery is a great challenge for the anesthesiologists. To keep the patients quiet, tranquil, pain-free and obeying verbal commands, with no complications during surgery, requires great skill to manipulate the doses of available sedative drugs and a good understanding of the surgical procedure.

Many studies[5–6] had evaluated the use of propofol and/or remifentanil as sedative drugs during the performance of the block (peri-retrobulbar) and for short ocular procedures.[7] However, sedation during the entire period of the prolonged ocular procedures was not adequately studied.

This study aims to evaluate the efficacy of the combined use of propofol and remifentanil as a sedative technique in comparison with the use of propofol alone to limit patient discomfort and movement during local anesthesia for vitreo-retinal surgery lasting for more than two hours.

## MATERIALS AND METHODS

This prospective randomized double blind study was carried out in Magrabi Eye and Ear Hospital, Muscat- Oman, between January 2006 and January 2008. After approval from the ethical committee and written informed consent from the patients, 140 patients were included in the study. The patients were scheduled for vitreo-retinal surgery, including vitrectomy, scleral buckle and cryopexy, under local anesthesia using peribulbar block with an expected surgical time of more than two hours.

Inclusion criteria included patients with ASA physical status I, II and III with no contraindications to local anesthesia or to the drugs used in this study. Exclusion criteria included; age younger than 40 years, operation time less than two hours, patients on aspirin or anticoagulants, uncontrolled hypertension, hyperthyroidism, frequent cough, drug abuse, impaired hearing, neurological or psychological disorders, and partial or failed block.

Patients were divided randomly using a closed envelop technique into two equal groups. Group I (study group) patients were given propofol bolus of 0.3 mg/kg followed by a continuous infusion of 0.3 to 0.5 mg/kg/h and a remifentanil bolus of 1 µg/kg over 30 seconds followed by a continuous infusion of 0.03-0.05 μg/kg/min, and group II (control group) patients were given propofol as in the first group+placebo (syringe was labeled as 20 μg/ ml similar to remifentanil syringe) with a bolus dose and a continuous infusion like that used for remifentanil in the first group. The sedative drugs were started 10 minutes before performance of the block and continued till the end of the surgical procedure. Propofol, remifentanil and placebo were administered continuously using ACM 5500 syringe pump (Beijing Aerospace Changfeng Co., Ltd, Medical devices branch, Beijing, China).

Titration of drugs was done with the aim to keep sedation at level 3 on the Ramsay Sedation Scale (patient responds to commands only). In the case of propofol after a bolus dose, the infusion was started at a rate of 0.3 mg/kg/h and increased every five minutes by 0.1 mg/kg/h to a maximum of 0.5 mg/kg/h. In case of remifentanil, after a bolus dose, the infusion was started at a rate of 0.03 μg/kg/min and increased gradually by 0.005 μg/kg/min every 5 minutes to a maximum of 0.05 μg/kg/min. After reaching the satisfactory level of sedation (3 on Ramsay Sedation Scale) the infusion rate was fixed unless there were signs of over-sedation like a respiratory rate of less than 10 per minute, SpO_2_ less than 95% or loss of verbal communication, where the rate of infusion was decreased in the same manner.

The patients were not pre-medicated and were kept fasting for eight hours before surgery. In the operating theatre, monitoring of ECG, non-invasive arterial blood pressure, respiratory rate and pulse oximetry was commenced using Insight 8500 informative patient monitor (Beijing Aerospace Changfeng Co., Ltd, Medical devices branch, Beijing, China). An intravenous catheter was inserted and Lactated Ringer’s solution was given at a rate of 4 ml/kg/h. Oxygen was given by nasal cannula at a rate of 4 l/min.

### Technique of peribulbar anesthesia used

Skin infiltration was done with 0.5 ml lidocaine using ½-inch 25-gauge needle at the junction of the medial two thirds and the lateral one-third of the lower eyelid. Then a one inch 25 gauge short bevel needle was inserted at the same site in a strictly posterior direction. The depth of insertion of the needle was limited to 25 mm. The local anesthetic solution (an equal mixture of 2% lignocaine and 0.5% bupivacaine and 30 IU/ml hyaluronidase) was injected after an aspiration test. The injected volume was not predetermined but adjusted to each patient. The injection continued until proptosis and lid fullness appeared with sensation of full orbit. Compression was applied for 15 to 20 minutes using a Honan’s balloon set at 40 mmHg to lower the intraocular pressure. If after 15 minutes, the degree of akinesia was not adequate, a second injection with the same anesthetic mixture was given in a similar way. The block was checked after another 10 minutes. If after the second injection, total akinesia was not achieved, the block was considered unsatisfactory. The procedure was converted to general anesthesia and the patient was excluded from the study.

All surgeries were done by the same surgeon who was blinded about the anesthetic medications used for sedation. The following measures were recorded by masked observers unaware of the anesthetic medications used.

1. Heart rate and non-invasive arterial blood pressure every 5 minutes from the time of start of administering sedative drugs till the end of the procedure.
2. Duration of surgery (from the time of surgical incision till the time of removal of surgical drapes).
3. Incidence of pain during the procedure which was assessed every 15 minutes using the verbal rating numerical pain score (0=no pain and 10 worst pain imaginable). The patients were made familiar with the use of this score before the surgery. A dose of 25 μg of fentanyl was given if the pain score became more than 5.
4. Incidence of discomfort using a discomfort score on a 10 point scale (where 0=none and 10=extreme discomfort) was assessed every 15 minutes. The patients were made familiar with the use of this score before the surgery. The patients were asked about the cause of any reported discomfort. The observer had to try to relieve the cause of discomfort and 25μg fentanyl was given, if the discomfort score became more than 5.
5. Total amount of fentanyl given as a rescue medicine during procedure.
6. The ophthalmologist -who was blinded to the sedation technique used-was asked to complete a Likert 5-point satisfaction scale[8] (with 1 representing the least and 5 the highest degree of satisfaction) with regard to patient condition during surgical procedure.
7. Incidence of patient movements during procedure according to the following scale:
  - No movement or movement with no effect on the surgical field.
  - Movement with a slight effect on the surgical field.
  - Movement with a moderate effect on the surgical field.
  - Movement with a major effect on the surgical field. NB. The effect on the surgical field was determined by the ability to keep the operated eye (cornea and sclera seen through eye speculum) under the microscope as seen from the monitor. A slight effect meant less than half of the eye outside the field, a moderate effect more than half of the eye outside the field and a major effect if the whole eye went outside the field.
8. Recording of any complications like nausea, vomiting, respiratory depression or obstruction.

Sample size was done using G*Power version 3.01.10 which indicated that 70 patients were required for each group. This was based on the data obtained from a pilot study done in the same institution which demonstrated a 25% incidence of movement and discomfort in propofol sedation with an anticipated reduction in the incidence of 50% which was our primary outcome. The alpha error was set at 0.05 and actual power at 95%. The statistical analysis of the present study was conducted through the computer program SPSS version 15.0 for windows. Data were expressed as mean±standard deviation (SD), or number (%). A two sided Chi-square and Fisher exact tests was used to compare qualitative variables, repeatedmeasures analysis of variance and unpaired t test were used for quantitative variables. A *P*-value less than 0.05 were considered statistically significant.

## RESULTS

The two groups were comparable with regard to age, weight, gender, ASA physical status and duration of surgery [Table 1].

**Table 1:** Demographic data and duration of surgery

	Group I N0.=70	Group II N0.=70
Age	60.9±5.6	62.8±6.7
Weight	64.9±9.9	65.4±13.9
Gender (M/F)	36/34	38/32
ASA physical status (I/II/III)	18/38/14	21/39/10
Duration of surgery (min.)	146.1±16	147.7±16.1

* Means significant

There was a significant decrease in heart rate and mean arterial blood pressure (MABP) in each group 10 minutes after the start of the sedation compared with pre-sedation data and continued all through the procedure [Table 2]. There was an insignificant difference between the two groups with regard to changes in heart rate and MABP all through surgical procedure [Table 2]. There was insignificant difference between the two groups with regard to the incidence of complications except for an increased incidence of breakthrough pain and discomfort which necessitated the use of fentanyl as a rescue treatment in the group II compared with group I *P*<0.001 [Table 3]. With regard to patient movements during the procedure, there were no instances of any movement with a major effect on the surgical field, which could affect surgical outcome, in either of the two groups. The number of patients who did not move was significantly higher in group I *P*<0.001 [Table 3]. The Ophthalmologist satisfaction scale was significantly higher in group I (4.5±0.63) compared with group II (3.7± 1.04) with *P*=0.0016.

**Table 2:** Changes in heart rate and mean arterial blood pressure

	Baseline	After 10 min	After 30 min	After 60 min	After 120 min
Heart rate (beats/min)					
Group I	77.2±17.5	68.5±13.9*	66.3±13.5*	67.8±12.6*	68.7±12.2*
Group II	81.5±15.8	72.1±11.9*	74.3±14*	76.6±11.8*	74.3±12.1*
MABP (mmHg)					
Group I	104±10.6	93.8±8.6*	86.5±7.4*	86.4 ±9.4*	83.8±8.4*
Group II	100.1±12.5	89.5±10.2*	83.7±9.4*	83±11.4*	80.2±9.4*

* Means significant within the same group compared with baseline;

@ Means significant between the two groups at the same time of measurement

**Table 3:** Incidence of complications and patient movements

	Group I no. (%)	Group II no. (%)
Incidence of pruritus	7 (10)	5 (7.1)
Incidence of breakthrough pain (pain score >5)	0 (0)	14 (20)*
Incidence of discomfort (Discomfort score >5)	3 (4.3)	18 (25.7)*
Incidence of movement		
No movement	56 (80)*	38 (54.29)
Movement with slight effect on surgical field	12 (17.14)	21 (30)*
Movement with moderate effect on surgical field	2 (2.86)*	11 (15.71)
Movement with major effect on surgical field	0 (0)	0 (0)
Number of patients who received fentanyl as a rescue treatment	3 (4.3)	29 (41.4)*
Total number of rescue fentanyl doses given per group	6	55*

* Means significant

## DISCUSSION

Anesthesia care for the ophthalmic surgical procedures under local anesthesia balances goals of patient comfort with safety and an optimal outcome. The results of this double-blind randomized study confirm the importance of adding analgesia to sedation in retinal surgery under local anesthesia lasting for more than two hours.

The combined use of propofol and remifentanil as a continuous infusion before performance of the block and during vitreo-retinal surgery was associated with a lower incidence of patient discomfort, breakthrough pain, and patient movement along with high degree of surgeons’ satisfaction and hemodynamic stability.

In this study, there was a significant decrease in heart rate and MABP in each group compared with pre-sedation level starting 10 minutes after the start of the sedation and continued all through the procedure till the end of surgery. This decrease could be attributed to the relief of anxiety and associated sympathetic over-activity caused by fear and apprehension because of surgery,[9–10] in addition, to the effect of propofol and remifentanil on vascular tone and autonomic nervous system. However, this decrease in heart rate and MABP was not significant clinically as it was less than 20% of pre-sedation values.[11]

The use of propofol alone as a sedative agent was not enough to deal with breakthrough pain or discomfort during the surgical procedure and fentanyl was given as a rescue treatment to relieve pain or discomfort. Adding remifentanil to propofol was effective in masking pain and discomfort during the surgical procedure. Holas *et al*.[7] reported in his study for sedation during eye surgery under retrobulbar block that superior pain relief was achieved with remifentanil used as a sole agent when compared with propofol. Rewari *et al*.[12] studied the use of various doses of remifentanil combined with propofol for sedation during placement of eye block. They found that the combination of remifentanil 0.5 μg/kg and propofol 0.5 mg/kg provided excellent anxiety and pain relief with the least adverse effects.

There were many causes of discomfort during the surgical procedure as reported by masked observers in this study: Some patients were not comfortable from lying down in the same position for a long period of time, or they had itching in their arms, face or they wanted to change the position of their heads due to pain in the head or neck. In addition, some patients wished to flex their legs due to pain. The incidence of discomfort was higher in the propofol group compared with remifentanil group, which could be explained by the potent analgesic effect of remifentanil.

The surgeon was satisfied with patient comfort and better surgical conditions as evidenced by reduced patient movement in the remifentanil group. In this study, we have not commented on patient response during performance of block as this has been studied before. Many studies have demonstrated that the combination of propofol and remifentanil was better than the use of propofol alone in limiting patient movement during performance of the block.[5–12]

The possibility of movement of the patients during surgery, which might affect the surgical outcome, could be considered as a point of weakness for the use of conscious sedation for ocular surgery. However, with proper selection and detailed explanation of the procedure to the patient, this risk could be avoided.

## CONCLUSION

The combined use of propofol and remifentanil as a continuous infusion before performance of the block and during lengthy vitreo-retinal surgery was associated with a lower incidence of patient discomfort, breakthrough pain, and patient movement along with high degree of surgeons’ satisfaction and hemodynamic stability.
